# Global burden of dengue from 1990 to 2021: a systematic analysis from the Global Burden of Disease study 2021

**DOI:** 10.1186/s40249-025-01365-x

**Published:** 2025-10-16

**Authors:** Jinxin Zheng, Hai Tong, Muxin Chen, Lei Duan, Peng Song, Jiahui Sun, Xiaonong Zhou, Xinyu Feng

**Affiliations:** 1 School of Global Health, Chinese Center for Tropical Diseases Research, Shanghai Jiao Tong University School of Medicine, Shanghai, 200025 China; 2 One Health Center, Shanghai Jiao Tong University, The University of Edinburgh, Shanghai, 200025 China; 3 National Institute of Parasitic Diseases, Chinese Center for Disease Control and Prevention (Chinese Center for Tropical Diseases Research); NHC Key Laboratory of Parasite and Vector Biology; WHO Collaborating Centre for Tropical Diseases, National Center for International Research On Tropical Diseases, Shanghai, 200025 China; 4 School of Life Science, Fudan University, Shanghai, 200438 People’s Republic of China

**Keywords:** Dengue fever, Global Burden of Disease, Incidence, Mortality, Disability-adjusted life years, Socio-demographic Index

## Abstract

**Background:**

Dengue fever remains a major global public health challenge, with increasing incidence and burden over recent decades. Global warming, urbanization, and increased international travel have fueled the global spread of dengue. Despite escalating global concern, recent and consistent estimates of the global dengue burden remain limited. This study aimed to quantify the shifting trends of dengue fever.

**Methods:**

We analyzed the 2021 Global Burden of Disease (GBD) dataset to assess dengue fever's incidence, prevalence, mortality, and disability-adjusted life years (DALYs) from 1990 to 2021 across 204 countries/regions. Data were stratified by age, sex, and socio-demographic index (SDI) using age-standardized rates, and time-trend analysis was conducted with general linear regression. Correlations between SDI and disease burden metrics were evaluated using Spearman’s rank correlation.

**Results:**

From 1990 to 2021, the global burden of dengue increased, with age-standardized incidence rate (ASIR) rising by 0.56% (95% UI: 0.23–2.38), age-standardized prevalence rate (ASPR) by 0.56% (95% UI: 0.23–2.36), and age-standardized DALYs rate (ASDR) by 0.28% (95% UI: -0.38–0.92). In 2021, there were an estimated 58.96 million cases. Regionally, Tropical Latin America reported the highest ASIR (5774.82; 95% UI: 1774.73–11,624.76). At the national level, variations in the change of the ASIR were observed across countries with Tonga reporting the highest ASIR in 2021. From 1990 to 2021, males exhibited a higher ASDR compared to females, particularly in the 0–14 age group. Dengue burden trends varied across SDI regions, with high-middle and middle SDI regions showing increased ASIR, while low SDI regions experienced a decline.

**Conclusions:**

The analysis highlights the increase in dengue burden globally, with demographic and geographic disparities. The findings underscore the need for targeted prevention, control, and treatment strategies to mitigate the growing burden of dengue fever worldwide.

**Supplementary Information:**

The online version contains supplementary material available at 10.1186/s40249-025-01365-x.

## Background

Dengue fever, an acute infectious disease caused by the dengue virus and transmitted primarily by *Aedes aegypti* and *Aedes albopictus* mosquitoes [1], represents a rapidly growing global health threat. Climate change [2,3], accelerated urbanization [4], and increased international travel [5] have fueled its expansion beyond traditional tropical regions into temperate zones [6,7]. This geographic spread is evidenced by local transmissions now documented in European countries including France [8] and Italy [6]. The World Health Organization estimates 50–100 million annual cases globally [9], though true incidence likely far exceeds reported numbers due to undiagnosed and asymptomatic infections [10]. Recent severe outbreaks across South America (Brazil [11], Argentina [12], Paraguay [13,14]) and Southeast Asia [15–17] highlight the escalating burden in endemic regions, with significant implications for public health systems and economies worldwide.

The dengue burden is immense and escalating, characterized by vast numbers of infections, significant morbidity, substantial economic costs, and a rapidly expanding geographic footprint. While previous studies have provided valuable insights into dengue burden, critical knowledge gaps persist. Existing analyses remain limited by outdated datasets, such as the most recent comprehensive estimates in 2019 [18], narrow geographic scope (e.g., country-specific or regional studies) [3,19], or methodological approaches that combine dengue with other vector-borne diseases [20,21]. Furthermore, few offer standardized stratification across age, sex, and socioeconomic indices that would enable global comparisons of burden disparities. The absence of contemporary, integrated analyses using unified metrics has hindered the development of targeted intervention strategies, leaving health systems without the granular data needed to optimize vector control, vaccine deployment, and clinical management in the face of accelerating dengue threats.

To address these gaps, we delved into the Global Burden of Disease (GBD) 2021 dataset to conduct a comprehensive 32-year longitudinal analysis (1990–2021) of dengue burden across 204 countries. Our study attempted to provide: 1) updated estimates of incidence, prevalence, mortality, and disability-adjusted life years (DALYs); 2) stratification by age, sex, and Socio-demographic Index (SDI) to identify vulnerable populations; and 3) temporal trend analysis reflecting post-2019 epidemiological shifts. These evidence-based insights will equip policymakers and health systems to optimize resource allocation and implement targeted control strategies for this expanding global threat.

## Methods

### Selection of data source and study design

The Global Burden of Disease (GBD) study, led by the Institute for Health Metrics and Evaluation (IHME) at the University of Washington, provides comprehensive annual estimates for health loss due to diseases and injuries, including dengue fever. The GBD 2021 database covers 204 countries and territories, 288 causes of death, 371 diseases and injuries, and 88 risk factors, providing a robust framework for analyzing epidemiological metrics such as incidence, prevalence, mortality, and Disability-Adjusted Life Years (DALYs).

This study conducted an advanced secondary data analysis using the GBD 2021 database to evaluate temporal trends in the global and regional burden of dengue fever from 1990 to 2021. Data were extracted from the Global Health Data Exchange (GHDx) query tool (http://ghdx.healthdata.org/gbd-results-tool), encompassing the general population across all age groups, genders, and SDI regions. As this study is a secondary data analysis utilizing aggregated data from the GBD 2021 database, there was no direct involvement of patients or the public in the research design, methodology, data collection, analysis, or dissemination plans.

### Disease burden metrics

Annual data were compiled on incident cases, prevalent cases, mortality counts, years of life lost (YLLs) due to premature mortality, years lived with disability (YLDs) accounting for non-fatal health outcomes, and disability-adjusted life years (DALYs), which integrate YLLs and YLDs into a single measure of overall disease burden. These metrics were stratified by age groups to capture variations in disease impact and by geographical regions, including seven super-regions, 21 sub-regions, and 204 countries. Countries were further categorized into SDI quintiles to assess disease burden dynamics across socio-economic contexts. Age-standardized incidence rates (ASIR), prevalence rates (ASPR), and death rates (ASDR) were computed using the GBD global reference population to ensure comparability across populations with different age structures. DALYs were calculated by summing YLLs and YLDs, with YLDs weighted by disability severity as per GBD methodology. The age-standardized rate (ASR) is calculated using the direct standardization method as follows:

$${\text{ASR}}=\frac{{\sum }_{i=1}^{A}{w}_{i}\times {r}_{i}}{{\sum }_{i=1}^{A}{w}_{i}}$$

where *A* is the total number of age groups, ${r}_{i}$ is the observed age-specific rate in age group i, ${w}_{i}$ is the number (or proportion) of individuals in age group i in the GBD world standard population.

Dengue cases were identified using International Classification of Diseases (ICD) codes, specifically ICD-10 codes A90–A91 for both non-fatal and fatal cases, and ICD-9 code 061 for historical data consistency. These codes ensured the inclusion of all relevant dengue fever cases while excluding unrelated conditions.

SDI quintiles, ranging from low to high, were defined by the GBD study based on the geometric mean of three scaled indices: total fertility rate for individuals under 25 years, average years of education for those aged 15 years and older, and lag-distributed income per capita. Each index is scaled to a 0–1 range, providing a composite measure of socio-economic development that facilitates comparisons across countries and regions.

### Statistical analysis

Epidemiological metrics for dengue incidence, prevalence, mortality, and DALYs were extracted from the GBD database, with age-standardization per 100,000 population using the GBD study's global reference. To assess temporal trends in dengue burden, we employed Joinpoint regression analysis to estimate average annual percent change (AAPC) in ASRs from 1990 to 2021. The Joinpoint model fits segmented log-linear regressions of the form:

$$\text{ln}\left({\text{ASR}}_{t}\right)={\upbeta }_{i}\cdot t+\alpha$$

where *t* denotes calendar year, and ${\beta }_{i}$ is the slope in segment *i*, corresponding to the Annual Percent Change (APC):

$${\text{APC}}i=\left[\text{exp}\left({\upbeta }_{i}\right)-1\right]\times 100\%$$

The AAPC was calculated as a weighted average of the APCs across all joinpoint segments during the specified interval (1990–2021), where weights correspond to the number of years within each segment:

$${\text{AAPC}}=\left[\text{exp}\left(\frac{{\sum }_{i=1}^{k}{w}_{i}\cdot {\upbeta }_{i}}{{\sum }_{i=1}^{k}{w}_{i}}\right)-1\right]\times 100\%$$

where ${w}_{i}$ is the duration of segment *i* within the interval of interest, and ${\sum }_{i=1}^{k}{w}_{i}$ is the total length of the time interval. Analyses were conducted at the global level and separately across GBD-defined regions.

Correlations between age-standardized DALY rates and SDI were assessed using Spearman’s rank correlation test, with statistical significance set at (*P* < 0.05). Given the focus on time trends and SDI correlations, multicollinearity was not a concern, as the primary independent variables (calendar year and SDI) are unlikely to be highly correlated. Locally weighted scatterplot smoothing was applied to visualize trends between SDI and age-standardized rates. All statistical analyses were conducted using R version 4.4.1 (R Foundation, Vienna, Austria).

## Results

### Global overview

In 2021, dengue fever affected an estimated 58,964,185 individuals globally (95% UI: 15,473,439–106,885,036), with an ASIR of 752.04 per 100,000 population (95% UI: 196.33–1,363.35). From 1990 to 2021, the ASIR rose by 0.56% (95% UI: 0.23–0.38). Regarding prevalence, there were 3,517,384 cases in 2021 (95% UI: 928,244–6,430,039), corresponding to an ASPR of 44.86 per 100,000 (95% UI: 11.77–82.13), an increase of 0.56 per 100,000 (95% UI: 0.23–2.36) since 1990. For DALYs, the ASDR increased from 21.63 per 100,000 in 1990 (95% UI: 15.09–26.92) to 27.76 in 2021 (95% UI: 14.21– 41.65), reflecting a percentage increase of 0.28 (95% UI: -0.38–0.92). However, the wide overlap in confidence intervals suggests that this rise may not be statistically significant. (Table 1, Supplementary File 1, Table S1).

**Table 1:** Global burden of dengue at global and regional level stratified by region and Sociodemographic Index (SDI), 1990–2021

	Incidence			Prevalence			DALYs		
	Age-standardised rate (per 100 ,000 population, 95%UI)		Percentage change of rates, 1990–2021 (95% UI)	Age-standardised rate (per 100,000 population, 95% UI)		Percentage change of rates, 1990–2021 (95%UI)	Age-standardised rate (per 100,000 population, 95% UI)		Percentage change of rates, 1990–2021 (95% UI)
	1990	2021		1990	2021		1990	2021	
Global	481.85 (70.76–946.29)	752.04 (196.33–1,363.35)	0.56 (0.23–2.38)	28.75 (4.23–57.77)	44.86 (11.77–82.13)	0.56 (0.23–2.36)	21.63 (15.09–26.92)	27.76 (14.21–41.65)	0.28 (-0.38–0.92)
Andean Latin America	305.85 (36.08–714.51)	593.22 (247.03–1,014.24)	0.94 (-0.19–18.65)	18.32 (2.10–43.68)	35.48 (14.70–63.09)	0.94 (-0.20–19.70)	3.58 (0.74–8.91)	6.56 (2.80–12.31)	0.83 (-0.15–9.10)
Australasia	28.69 (1.996–84.63)	58.99 (18.09–139.69)	1.06 (-0.59–42.59)	1.71 (0.12–5.49)	3.53 (0.97–9.25)	1.06 (-0.60–43.06)	0.30 (0.02–0.99)	0.61 (0.16–1.60)	1.08 (-0.59–41.37)
Caribbean	416.07 (13.34–1220.54)	475.93 (60.68–1,325.33)	0.14 (-0.46–10.87)	24.79 (0.77–74.65)	28.49 (3.49–80.01)	0.15 (-0.50–10.75)	5.02 (0.84–14.80)	5.83 (1.17–15.48)	0.16 (-0.42–2.07)
Central Asia	0.00 (0.00–0.00)	0.00 (0.00–0.00)	0.00 (0.00–0.00)	0.00 (0.00–0.00)	0.00 (0.00–0.00)	0.00 (0.00–0.00)	0.00 (0.00–0.00)	0.00 (0.00–0.00)	NA
Central Europe	0.00 (0.00–0.00)	0.00 (0.00–0.00)	0.00 (0.00–0.00)	0.00 (0.00–0.00)	0.00 (0.00–0.00)	0.00 (0.00–0.00)	0.00 (0.00–0.00)	0.00 (0.00–0.00)	NA
Central Latin America	696.38 (115.55–1409.02)	1140.37 (694.53–1599.48)	0.64 (-0.09–7.61)	41.51 (6.89–85.01)	67.95 (40.54–97.63)	0.64 (-0.09–7.32)	8.26 (2.37–17.68)	17.53 (9.92–27.60)	1.12 (0.31–5.15)
Central Sub-Saharan Africa	133.39 (1.64–850.51)	178.08 (8.62–1019.40)	0.34 (-0.85–13.49)	8.02 (0.10–45.52)	10.86 (0.50–59.03)	0.35 (-0.85–13.46)	1.36 (0.02–8.41)	1.91 (0.09–10.31)	0.40 (-0.84–11.35)
East Asia	3.03 (0.30–10.15)	4.27 (1.11–10.66)	0.41 (-0.60–12.14)	0.18 (0.02–0.56)	0.25 (0.07–0.62)	0.40 (-0.61–12.00)	0.34 (0.20–0.49)	0.09 (0.04–0.18)	-0.72 (-0.84–0.53)
Eastern Europe	0.00 (0.00–0.00)	0.00 (0.00–0.00)	0.00 (0.00–0.00)	0.00 (0.00–0.00)	0.00 (0.00–0.00)	0.00 (0.00–0.00)	0.00 (0.00–0.00)	0.00 (0.00–0.00)	NA
Eastern Sub-Saharan Africa	829.74 (63.78–2162.22)	94.37 (1.55–364.39)	-0.89 (-1.00–2.86)	49.41 (3.77–133.14)	5.59 (0.09–21.71)	-0.89 (-1.00–2.82)	8.17 (0.70–21.51)	1.28 (0.23–4.12)	-0.84 (-0.99–2.46)
High-income Asia Pacific	130.09 (19.12–340.20)	294.01 (65.07–679.53)	1.26 (-0.20–12.48)	7.77 (1.07–21.24)	17.60 (3.54–43.36)	1.27 (-0.20–12.32)	1.30 (0.17–3.53)	2.90 (0.52–7.39)	1.23 (-0.21–10.12)
High-income North America	0.10 (0.02–0.35)	0.36 (0.01–1.86)	2.45 (-0.87–24.52)	0.01 (0.00–0.02)	0.02 (0.00–0.10)	2.40 (-0.88–22.93)	0.00 (0.00–0.01)	0.02 (0.01–0.04)	4.27 (1.02–10.29)
North Africa and Middle East	5.23 (1.74–16.53)	8.50 (2.86–30.02)	0.63 (-0.65–6.68)	0.31 (0.10–1.00)	0.51 (0.17–1.83)	0.63 (-0.61–6.98)	0.25 (0.15–0.41)	0.18 (0.08–0.45)	-0.27 (-0.68–0.86)
Oceania	370.69 (81.90–969.58)	486.03 (211.17–953.78)	0.31 (-0.45–3.55)	21.98 (4.85–56.47)	28.95 (12.35–58.06)	0.32 (-0.45–3.45)	13.43 (8.60–20.83)	6.04 (2.73–11.88)	-0.55 (-0.73–0.24)
South Asia	1,163.51 (12.61–2478.46)	1726.94 (102.48–3635.94)	0.48 (0.21–12.09)	69.58 (0.73–150.95)	103.22 (5.90–220.59)	0.48 (0.20–12.59)	35.79 (17.97–57.41)	53.46 (19.54–91.91)	0.49 (-0.14–1.03)
Southeast Asia	584.49 (114.66–1585.42)	971.89 (691.33–1500.41)	0.66 (-0.45–9.18)	34.71 (6.68–96.33)	57.78 (40.63–91.25)	0.66 (-0.47–9.15)	134.99 (88.76–204.74)	147.04 (95.32–200.97)	0.09 (-0.49–0.91)
Southern Latin America	77.21 (1.62–267.93)	118.83 (26.67–278.96)	0.54 (-0.50–61.62)	4.59 (0.09–16.23)	7.07 (1.49–16.92)	0.54 (-0.50–60.51)	0.81 (0.03–3.05)	1.22 (0.22–3.28)	0.51 (-0.51–35.45)
Southern Sub-Saharan Africa	1.23 (0.06–8.10)	1.32 (0.07–8.83)	0.07 (-0.96–31.56)	0.07 (0.00–0.50)	0.08 (0.00–0.52)	0.04 (-0.96–31.62)	0.02 (0.00–0.09)	0.01 (0.00–0.10)	-0.02 (-0.91–9.23)
Tropical Latin America	4,460.04 (265.15–11,852.45)	5774.82 (1774.73–11,624.76)	0.29 (-0.27–9.25)	265.61 (15.64–691.39)	343.77 (107.96–700.31)	0.29 (-0.26–9.18)	43.56 (3.32–113.62)	63.76 (22.43–136.04)	0.46 (-0.17–8.34)
Western Europe	0.00 (0.00–0.00)	0.00 (0.00–0.00)	0.00 (0.00–0.00)	0.00 (0.00–0.00)	0.00 (0.00–0.00)	0.00 (0.00–0.00)	0.02 (0.01–0.03)	0.00 (0.00–0.00)	-0.99 (-1.00–0.97)
Western Sub-Saharan Africa	378.12 (0.22–1,357.39)	512.53 (25.35–1792.58)	0.36 (-0.48–270.36)	22.50 (0.01–78.76)	30.38 (1.51–111.89)	0.35 (-0.46–271.80)	3.68 (0.02–14.27)	4.93 (0.24–17.05)	0.34 (-0.46–32.59)
SDI index									
High SDI	38.03 (5.47–92.83)	54.64 (12.22–119.21)	0.44 (-0.33–6.49)	2.27 (0.30–5.75)	3.27 (0.69–7.59)	0.44 (-0.34–6.23)	0.42 (0.09–1.07)	0.58 (0.14–1.39)	0.38 (-0.33–3.73)
High-middle SDI	98.43 (12.21–263.08)	215.66 (91.61–372.30)	1.19 (-0.05–15.17)	5.84 (0.69–16.18)	12.79 (5.04–23.03)	1.19 (-0.09–15.40)	8.92 (6.20–12.61)	11.39 (6.67–16.03)	0.28 (-0.42–1.13)
Middle SDI	782.97 (51.81–1,725.40)	1269.27 (437.36–2268.00)	0.62 (0.08–10.11)	46.71 (3.05–102.30)	75.70 (25.59–136.59)	0.62 (0.07–10.46)	33.64 (21.46–42.85)	48.78 (27.32–71.02)	0.45 (-0.26–1.31)
Low-middle SDI	802.75 (79.39–1725.04)	1117.70 (123.23–2373.55)	0.39 (-0.22–1.16)	47.95 (4.73–101.66)	66.77 (7.37–141.34)	0.39 (-0.22–1.10)	35.92 (24.95–47.94)	43.35 (19.69–69.74)	0.21 (-0.41–0.78)
Low SDI	431.07 (242.88–648.51)	368.03 (11.69–884.71)	-0.15 (-0.97–1.07)	25.64 (14.03–39.36)	21.87 (0.68–54.56)	-0.15 (-0.97–1.08)	11.84 (8.11–16.08)	12.34 (4.44–22.08)	0.04 (-0.61–0.82)

*DALYs* Disability-adjusted life years, *UI* Uncertainty interval

### Regional level

At the regional level, Tropical Latin America recorded the highest ASIR in 2021, at 5,774.82 per 100,000 population (95% UI: 1,774.73–11,624.76), with over 13.04 million incident cases (95% UI: 3,996,126–26,305,622). South Asia followed with an ASIR of 1,726.94 per 100,000 (95% UI: 102.48–3,635.94). These rankings align with those from 1990 (Supplementary File 2, Fig. S1). In terms of prevalence, Tropical Latin America led with an ASPR of 343.77 per 100,000 (95% UI: 107.96–700.31), followed by South Asia and Central Latin America. For DALYs, Southeast Asia had the highest ASDR in 2021 at 147.04 per 100,000 (95% UI: 95.32–200.97), with Tropical Latin America and South Asia also showing elevated rates. In contrast, high-income regions like Europe and North America reported the lowest ASDRs (Table 1).

### National level

Nationally, ASIR varied widely from 1990 to 2021. Tonga had the highest ASIR in 2021, at 14,363 per 100,000 (95% UI: 136–50,917), followed by Seychelles, Comoros, Marshall Islands, Singapore, and Cabo Verde. In 1990, Comoros topped the list with an ASIR of 11,154.07 per 100,000 (95% UI: 1,653.69–44,214.37). For prevalence, Tonga reported the highest ASPR in 2021, followed by Seychelles and Comoros. Regarding DALYs, Indonesia had the highest ASDR in 2021 at 279.79 per 100,000 (95% UI: 170.92–404.43), with Tonga and the Philippines also notable. Detailed trends are available in Supplementary File 4, Table S2.

### Temporal trends by SDI

**Fig. 1:**
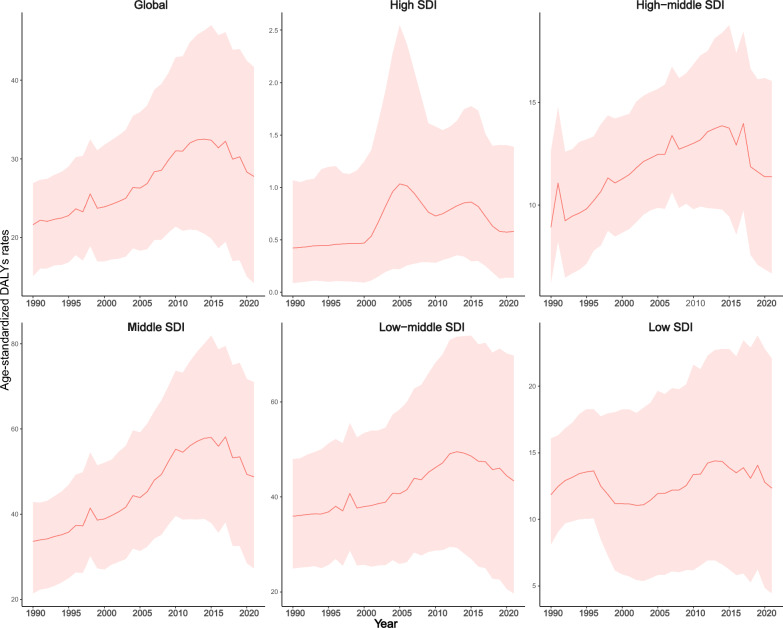
Temporal Trends of dengue burden by age-standardized DALYs rates (ASDR), both sexes combined, 1990–2021. DALYs: disability-adjusted life years

Globally, ASIR increased from 1990 to 2021, particularly in high-middle and middle SDI regions. In high-middle SDI regions, ASIR rose from 98.43 per 100,000 in 1990 (95% UI: 12.22–263.08) to 215.66 in 2021 (95% UI: 91.61–372.30). Middle SDI regions saw a similar rise, from 782.97 (95% UI: 51.81–1725.40) to 1269.27 per 100,000 (95% UI: 437.36–2268.00) (Supplementary File 5, Table S3). Conversely, low SDI regions experienced a decline, from 431.07 (95% UI: 242.88–648.05) to 368.03 per 100,000 (95% UI: 11.69–884.71). In terms of prevalence, ASPR increased in most regions but decreased in low SDI areas. For DALYs, ASDR trends were similar across high-middle and middle SDI regions, while high SDI regions peaked at 1.03 per 100,000 in 2005 (95% UI: 0.22–2.55). Low SDI regions remained stable. These patterns indicate that dengue burden does not strictly correlate with SDI, possibly due to factors like urbanization and climate change (Fig. 1).

### Age and sex patterns

**Fig. 2:**
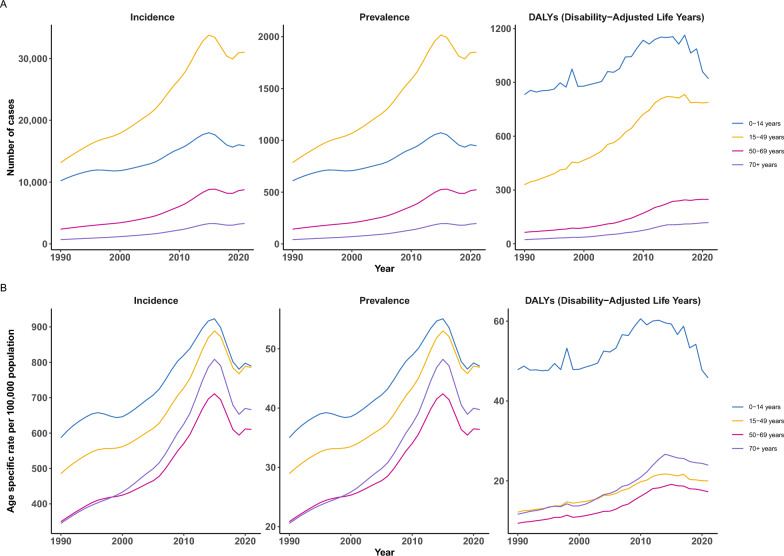
Trends in disease incidence, prevalence, disability-adjusted life years (DALYs), and age-specific rates per 100,000 population across age groups and time periods: 1990–2020. A: the trends of dengue burden indicated by incident cases, prevalent cases and DALYs, B: the trends of dengue burden indicated by age-standardized incidence rates (ASIR), prevalence rate (ASPR) and DALYs rates (ASDR)

**Fig. 3:**
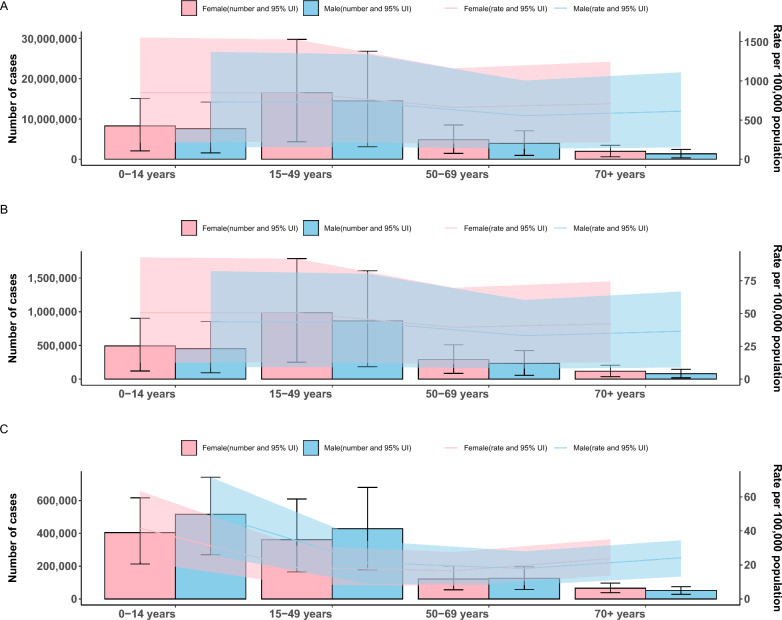
The dengue burden for male and female attributed to dengue in 2019. A: the dengue burden for male and female indicated by incident cases and age-standardized incidence rates (ASIR), B: the dengue burden for male and female indicated by prevalent cases and prevalence age-standardized rate (ASPR), C: the dengue burden for male and female indicated by DALY and age-standardized rate DALYs rates (ASDR). DALYs: disability-adjusted life years

From 1990 to 2021, dengue incidence and prevalence rates rose across all age groups until 2015, followed by a notable decline (Fig. 2). The 15–49 age group had the highest number of incident and prevalent cases (Fig. 3A), while the 0–14 group showed higher rates per 100,000 (Fig. 3B). The 0–14 group also had the highest ASDR, with fluctuations over time.

Females exhibited slightly higher ASIR and ASPR than males from 1990 to 2021, but males had a higher ASDR (Supplementary File 7, Fig. S3). In 2021, females accounted for 31.6 million cases, compared to 27.3 million for males.

Among females, the 15–49 age group had the most cases (16,530,266; 95% UI: 4,357,395–29,724,055), with an incidence rate of 848.20 per 100,000 (95% UI: 223.59–1525.21), closely followed by the 0–14 age group at 849.66 per 100,000 (95% UI: 211.05–1,552.60) (Fig. 3A, B). For males, the 0–14 age group had 7,607,459 cases, with an incidence rate of approximately 732.78 per 100,000 (95% UI: 151.01–1,368.16). In terms of prevalence, females aged 15–49 led in case numbers, while the 0–14 age group had the highest rate per 100,000. Males aged 50 and above consistently showed the lowest rates (Supplementary File 8, Table S5; Supplementary File 9, Table S6). For DALYs, males in the 0–14 age group had a notably high ASDR (Fig. 3C).

### Relationship between DALYs and SDI

**Fig. 4:**
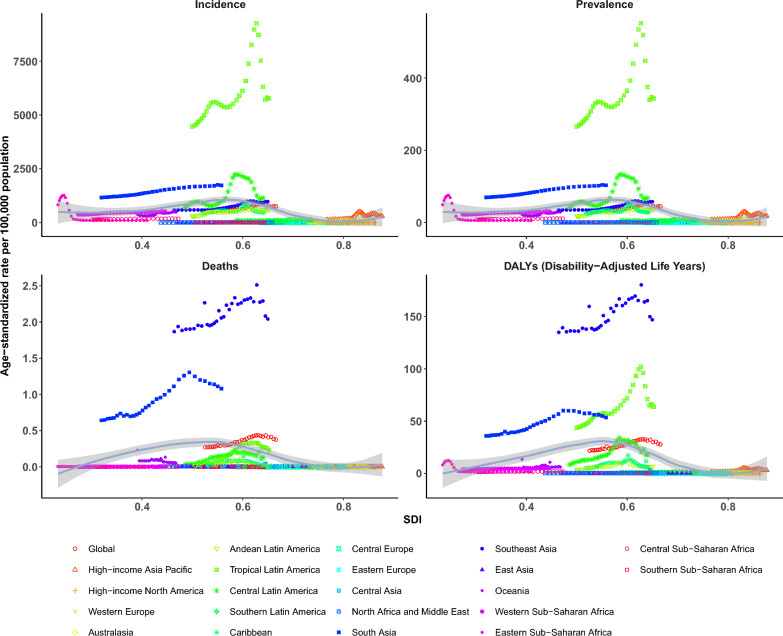
The association between SDI and age-standardized DALYs rates (ASDR) of dengue by 21 GBD regions and 204 countries and territories, both sexes combined, 2021. DALYs: disability-adjusted life years, SDI: Socio-demographic index

Analysis across 204 countries/regions showed a non-linear relationship between ASIR, ASPR, ASDR, and SDI. High ASIR was not exclusive to low-SDI regions; countries like Tonga, Seychelles, Comoros, Marshall Islands, and Singapore exceeded expectations based on SDI rankings, while the United States and South Sudan fell below. Similarly, ASPR and ASDR showed no consistent negative correlation with SDI. Nations such as Tonga, the Philippines, Comoros, and Seychelles had higher-than-expected ASDR, whereas Spain and Greece had lower rates than predicted (Fig. 4, Supplementary File 10, Fig. S4). This suggests that factors like climate, urbanization, and vector control, beyond socio-economic development, significantly influence dengue burden.

## Discussion

This study provides a comprehensive analysis of the global, regional, and national burden of dengue from 1990 to 2021. Our findings reveal a persistent and escalating global health challenge characterized by a significant increase in the absolute number of cases and a complex, shifting epidemiological landscape. The global ASIR of dengue has continued its upward trajectory over the past three decades, a trend consistent with previous reports that have documented the disease's relentless expansion [20,22]. This growth is driven by a confluence of factors, including climate change, rapid and unplanned urbanization, and increased global travel and trade [23,24].

Our analysis highlights profound geographical disparities in the dengue burden. The concentration of high incidence, prevalence, and DALYs in Tropical Latin America, South Asia, and Southeast Asia aligns with the known endemicity in these regions [25,26]. Countries in these areas, such as Brazil [3,27] and Vietnam [28] have long struggled with endemic and epidemic dengue. A particularly striking finding is the disproportionately high ASIR in several small island nations, including Tonga, Seychelles, and the Marshall Islands. This vulnerability may be attributed to immunologically naive populations, small population size, and the profound impact of imported cases via international travel, which can quickly ignite large-scale outbreaks in contained environments [29].

The relationship between dengue burden and the SDI is complex and non-linear. Our results show that the most significant increases in ASIR occurred in middle and high-middle SDI regions, a pattern also observed in other studies [22]. This suggests that economic development, particularly when accompanied by rapid, often poorly planned urbanization, can create conditions that favor vector proliferation and disease transmission. Conversely, the apparent decrease in ASIR in low-SDI regions should be interpreted with extreme caution. This is more likely a reflection of weak surveillance systems, limited diagnostic capacity, and significant under-reporting rather than a true decline in disease activity [20]. The relatively stable but persistent DALYs in these low-SDI nations further suggest that when dengue does occur, it may lead to more severe outcomes due to limited access to healthcare.

Our analysis of age and sex patterns provides critical insights for public health targeting. The highest incidence and prevalence rates were observed in the 0–14 age group, confirming that children remain a key vulnerable population, a finding supported by clinical studies in high-burden areas [27]. This age group also bears a high DALY burden, reflecting the long-term impact of premature mortality and disability. However, the 15–49 age group accounted for the largest absolute number of cases, highlighting the significant socioeconomic impact of dengue on the most economically productive segment of the population. The slightly higher incidence in females may relate to differential exposure patterns or healthcare-seeking behaviors, while the higher DALY rate in males warrants further investigation into potential differences in disease severity or risk factors. The observed decline in burden across all age groups after 2015 is an intriguing trend that could be linked to intensified vector control efforts prompted by the Zika virus epidemic. However, recent reports of massive outbreaks in countries like Bangladesh [30] and Brazil suggest this reprieve may have been temporary, underscoring the volatile, epidemic-prone nature of dengue.

This study has several strengths, including its use of a comprehensive, standardized dataset from the GBD 2021 study, which allows for robust comparisons of trends over three decades across diverse geographical and socioeconomic settings. The analysis by age, sex, and SDI provides a granular view of the disease's epidemiology. However, several limitations must be acknowledged. First, the study is reliant on modeled data, and its accuracy is contingent on the quality and availability of primary data from individual countries. Dengue is notoriously under-reported due to a high proportion of asymptomatic infections, misdiagnosis as other febrile illnesses, and variable surveillance system capacities [31]. This limitation is particularly relevant for low-SDI regions and likely leads to an underestimation of the true burden. Second, the GBD data does not differentiate between the four dengue serotypes, which is a critical factor in determining epidemic potential and the risk of severe disease [32]. Finally, the data extends only to 2021, missing the major resurgence of dengue in several parts of the world in subsequent years.

The findings of this study have significant implications for global public health. The unabated rise of dengue, particularly in middle-income countries, demands a paradigm shift from reactive outbreak response to proactive, sustained prevention and control. This requires strengthening integrated vector management, improving urban sanitation, and enhancing public awareness. Furthermore, surveillance systems must be fortified globally. This includes expanding laboratory diagnostic capabilities to reduce misdiagnosis, standardizing reporting, and developing sophisticated early warning systems that integrate epidemiological data with climate and environmental predictors [33,34]. Future research should prioritize understanding the drivers behind the shifting epidemiology, including the complex interplay between socioeconomic development and disease risk. Evaluating the real-world, long-term impact of novel interventions, such as the deployment of *Wolbachia*-infected mosquitoes [35] and the rollout of new vaccines, will be crucial for shaping future control strategies.

## Conclusions

Dengue continues to be a formidable and expanding global health threat. Our study maps its extensive burden and highlights the evolving demographic and socioeconomic contours of its impact. While the global health community has made progress in understanding this complex disease, the wide uncertainty intervals in burden estimates and the persistent gaps in surveillance underscore the urgent need for greater investment in research, prevention, and control to mitigate the impact of dengue in an increasingly interconnected and environmentally vulnerable world.

## Supplementary Information

**Additional file 1: Table S1.** Global and regional burden of dengue: incidence, prevalence, and DALYs from 1990 to 2021.

**Additional file 2: Fig S1.** Global distribution of dengue burden measured by age-standardized incidence rates (ASIR) across regions and nations, 2021.

**Additional file 3: Fig S2.** Age-standardized prevalence rates by geographical regions.

**Additional file 2: Table S2.** Age-standardized DALYs rate of Dengue per 100,000 population in 2021 by country.

**Additional file 5: Table S3.** Age-standardized incidence rates of Dengue per 100,000 population by sociodemographic index (five categories; countries with a high, high-middle, middle, low-middle, or low sociodemographic index) from 1990 to 2019.

**Additional file 6: Table S4.** Age-standardized prevalence rates of Dengue per 100,000 population by sociodemographic index (five categories; countries with a high, high-middle, middle, low-middle, or low sociodemographic index) from 1990 to 2019.

**Additional file 7: Fig S3.** Temporal trends of incidence, prevalence, and DALYs (both males and females separately) from 1990 to 2020.

**Additional file 8: Table S5.** Age-standardized incidence rates of Dengue per 100,000 population by age and sex, 2021.

**Additional file 9: Table S6.** Age-standardized prevalence rates of Dengue per 100,000 population by age and sex, 2021.

**Additional file 10: Fig S4.** Relationship between SDI and Age-Standardized Rates across countries, colored by their respective Socio-demographic Index (SDI).

## Data Availability

Data used in the analyses can be obtained from the Global Health Data Exchange Global Burden of Disease Results Tool(https://ghdx.healthdata.org/gbd-results-tool).

## Abbreviations

ASIR: Age-standardized incidence rate
ASPR: Age-standardized prevalence rate
ASDR: Age-standardized DALYs rate
DALYs: Disability-adjusted life years
YLLs: Years of life lost
YLDs: Years lived with disability
SDI: Socio-demographic index
GBD: Global Burden of Disease
IHME: Institute for Health Metrics and Evaluation
GHDx: Global Health Data Exchange
ICD: International Classification of Diseases
AAPC: Average annual percent change
APC: Annual percent change
UI: Uncertainty interval
ASR: Age-standardized rate

## References

[1] Wilder-Smith A, Lindsay SW, Scott TW, Ooi EE, Gubler DJ, Das P. The Lancet Commission on dengue and other Aedes-transmitted viral diseases. Lancet. 2020;395(10241):1890–1. 32563358 10.1016/S0140-6736(20)31375-1

[2] Pandey BD, Costello A. The dengue epidemic and climate change in Nepal. Lancet. 2019;394(10215):2150–1. 31839187 10.1016/S0140-6736(19)32689-3

[3] Araújo VEM, Bezerra JMT, Amâncio FF, Passos VMA, Carneiro M. Increase in the burden of dengue in Brazil and federated units, 2000 and 2015: analysis of the Global Burden of Disease Study 2015. *Rev Bras Epidemiol* 2017, 20Suppl 01(Suppl 01):205–216. 10.1590/1980-5497201700050017 28658384

[4] Wu PC, Lay JG, Guo HR, Lin CY, Lung SC, Su HJ. Higher temperature and urbanization affect the spatial patterns of dengue fever transmission in subtropical Taiwan. Sci Total Environ. 2009;407(7):2224–33. 19157509 10.1016/j.scitotenv.2008.11.034

[5] Ratnam I, Leder K, Black J, Torresi J. Dengue fever and international travel. J Travel Med. 2013;20(6):384–93. 24165383 10.1111/jtm.12052

[6] Cassaniti I, Ferrari G, Senatore S, Rossetti E, Defilippo F, Maffeo M, et al. Preliminary results on an autochthonous dengue outbreak in Lombardy Region, Italy, August 2023. Euro Surveill. 2023;28:37. 10.2807/1560-7917.ES.2023.28.37.2300471 PMC10687988 37707980

[7] Tomasello D, Schlagenhauf P. Chikungunya and dengue autochthonous cases in Europe, 2007–2012. Travel Med Infect Dis. 2013;11(5):274–84. 23962447 10.1016/j.tmaid.2013.07.006

[8] Eldin C, Gautret P, Nougairede A, Sentis M, Ninove L, Saidani N, et al. Identification of dengue type 2 virus in febrile travellers returning from Burkina Faso to France, related to an ongoing outbreak, October to November 2016. Euro Surveill. 2016;21:50. 10.2807/1560-7917.ES.2016.21.50.30425 PMC5291134 28006651

[9] Bhatt S, Gething PW, Brady OJ, Messina JP, Farlow AW, Moyes CL, et al. The global distribution and burden of dengue. Nature. 2013;496(7446):504–7. 23563266 10.1038/nature12060 PMC3651993

[10] Wilder-Smith A, Byass P. The elusive global burden of dengue. Lancet Infect Dis. 2016;16(6):629–31. 26874620 10.1016/S1473-3099(16)00076-1

[11] Navarro JC, Arrivillaga-Henríquez J, Salazar-Loor J, Rodriguez-Morales AJ. COVID-19 and dengue, co-epidemics in Ecuador and other countries in Latin America: pushing strained health care systems over the edge. Travel Med Infect Dis. 2020;37:101656. 32268196 10.1016/j.tmaid.2020.101656 PMC7130119

[12] Rapaport S, Mauriño M, Morales MA, Fabbri C, Luppo V, Buyayisqui MP, et al. Epidemiology of dengue in Argentina during the 2010/11 to 2019/20 seasons: a contribution to the burden of disease. Trop Med Infect Dis. 2024. 10.3390/tropicalmed9020045. 38393134 10.3390/tropicalmed9020045 PMC10891897

[13] Gomez REG. 1475Effects of climate factors on the dengue fever in Paraguay: generalized additive model in 2014–2020. *International Journal of Epidemiology* 2021, 50(Supplement_1).

[14] Fonseca SNS. Changing epidemiology of dengue fever in children in South America. Curr Opin Pediatr. 2023;35(2):147–54. 36715049 10.1097/MOP.0000000000001220

[15] Phadungsombat J, Vu HTT, Nguyen QT, Nguyen HTV, Nguyen HTN, Dang BT, et al. Molecular characterization of dengue virus strains from the 2019–2020 epidemic in Hanoi, Vietnam. Microorganisms. 2023. 10.3390/microorganisms11051267. 37317240 10.3390/microorganisms11051267 PMC10222920

[16] L’Azou M, Moureau A, Sarti E, Nealon J, Zambrano B, Wartel TA, et al. Symptomatic dengue in children in 10 Asian and Latin American countries. N Engl J Med. 2016;374(12):1155–66. 27007959 10.1056/NEJMoa1503877

[17] Nontapet O, Maneerattanasak S, Jaroenpool J, Phumee A, Krachai W, Napet P, et al. Understanding dengue solution and larval indices surveillance system among village health volunteers in high- and low-risk dengue villages in southern Thailand. One Health. 2022;15:100440. 36277094 10.1016/j.onehlt.2022.100440 PMC9582563

[18] Ilic I, Ilic M. Global patterns of trends in incidence and mortality of dengue, 1990–2019: an analysis based on the Global Burden of Disease Study. Medicina (Kaunas). 2024. 10.3390/medicina60030425. 38541151 10.3390/medicina60030425 PMC10972128

[19] Anker M, Arima Y. Male-female differences in the number of reported incident dengue fever cases in six Asian countries. Western Pac Surveill Response J. 2011;2(2):17–23. 23908884 10.5365/WPSAR.2011.2.1.002 PMC3730962

[20] Zeng Z, Zhan J, Chen L, Chen H, Cheng S. Global, regional, and national dengue burden from 1990 to 2017: a systematic analysis based on the global burden of disease study 2017. EClinicalMedicine. 2021;32:100712. 33681736 10.1016/j.eclinm.2020.100712 PMC7910667

[21] Liang Y, Dai X. The global incidence and trends of three common flavivirus infections (dengue, yellow fever, and zika) from 2011 to 2021. Front Microbiol. 2024;15:1458166. 39206366 10.3389/fmicb.2024.1458166 PMC11349664

[22] Yang X, Quam MBM, Zhang T, Sang S. Global burden for dengue and the evolving pattern in the past 30 years. J Travel Med. 2021. 10.1093/jtm/taab146. 34510205 10.1093/jtm/taab146

[23] Findlater A, Bogoch II. Human mobility and the global spread of infectious diseases: a focus on air travel. Trends Parasitol. 2018;34(9):772–83. 30049602 10.1016/j.pt.2018.07.004 PMC7106444

[24] Fèvre EM, Bronsvoort BM, Hamilton KA, Cleaveland S. Animal movements and the spread of infectious diseases. Trends Microbiol. 2006;14(3):125–31. 16460942 10.1016/j.tim.2006.01.004 PMC7119069

[25] Zhang WX, Zhao TY, Wang CC, He Y, Lu HZ, Zhang HT, et al. Assessing the global dengue burden: Incidence, mortality, and disability trends over three decades. PLoS Negl Trop Dis. 2025;19(3):e0012932. 40072961 10.1371/journal.pntd.0012932 PMC11925280

[26] Tian N, Zheng JX, Guo ZY, Li LH, Xia S, Lv S, et al. Dengue incidence trends and its burden in major endemic regions from 1990 to 2019. Trop Med Infect Dis. 2022. 10.3390/tropicalmed7080180. 36006272 10.3390/tropicalmed7080180 PMC9416661

[27] Ricardo-Rivera SM, Aldana-Carrasco LM, Lozada-Martinez ID, Bolaño-Romero MP, Acevedo-Lopez N, Sajona-Leguia WA, et al. Mapping dengue in children in a Colombian Caribbean Region: clinical and epidemiological analysis of more than 3500 cases. Le Infez Med. 2022;30(4):602–9. 10.53854/liim-3004-16 PMC9715006 36482961

[28] Cuong HQ, Hien NT, Duong TN, Phong TV, Cam NN, Farrar J, et al. Quantifying the emergence of dengue in Hanoi, Vietnam: 1998–2009. PLoS Negl Trop Dis. 2011;5(9):e1322. 21980544 10.1371/journal.pntd.0001322 PMC3181236

[29] Kaiwan O, Sethi Y, Khehra N, Padda I, Chopra H, Chandran D, et al. Emerging and re-emerging viral diseases, predisposing risk factors, and implications of international travel: a call for action for increasing vigilance and imposing restrictions under the current threats of recently emerging multiple Omicron subvariants. International journal of surgery (London, England). 2023;109(3):589–91. 37093096 10.1097/JS9.0000000000000176 PMC10389581

[30] Mahmud AS, Bhattacharjee J, Baker RE, Martinez PP. Alarming trends in dengue incidence and mortality in Bangladesh. J Infect Dis. 2024;229(1):4–6. 38000901 10.1093/infdis/jiad529 PMC10786241

[31] Yousuf R, Salam M, Akter S, Sinha S, Haque M. Dengue Dynamics: A Global Update. *Adv Hum Biol *2023.

[32] Nafisa T, Akram A, Yeasmin M, Islam Resma T, Siddique MAB, Hosen N, et al. Predominant dengue virus serotype in Dhaka, Bangladesh: a research letter on samples from 2022 outbreak. Health Sci Rep. 2024;7(1):e1818. 38250477 10.1002/hsr2.1818 PMC10797646

[33] Kesetyaningsih T, Kusbaryanto K, Widayani P. Dengue hemorrhagic fever prediction in coastal area using geographically weighted regression. Int J Public Health Sci. 2024;13(2):715–715.

[34] Hii YL, Zhu H, Ng N, Ng LC, Rocklöv J. Forecast of dengue incidence using temperature and rainfall. PLoS Negl Trop Dis. 2012;6(11):e1908. 23209852 10.1371/journal.pntd.0001908 PMC3510154

[35] Hoffmann AA, Ahmad NW, Keong WM, Ling CY, Ahmad NA, Golding N, et al. Introduction of *Aedes aegypti* mosquitoes carrying wAlbB *Wolbachia* sharply decreases dengue incidence in disease hotspots. iScience. 2024;27(2):108942. 38327789 10.1016/j.isci.2024.108942 PMC10847733

