# Supplementary material for: Global burden of dengue from 1990 to 2021: a systematic analysis from the Global Burden of Disease study 2021
Source: Infect Dis Poverty. 2025 Oct 16;14:105. doi: 10.1186/s40249-025-01365-x (PMC12529819; doi:10.1186/s40249-025-01365-x)
Supplement: Supplementary file 2 — Additional file 2: Fig S1. Global distribution of dengue burden measured by age-standardized incidence rates (ASIR) across regions and nations, 2021. [file 40249_2025_1365_MOESM2_ESM.pdf]

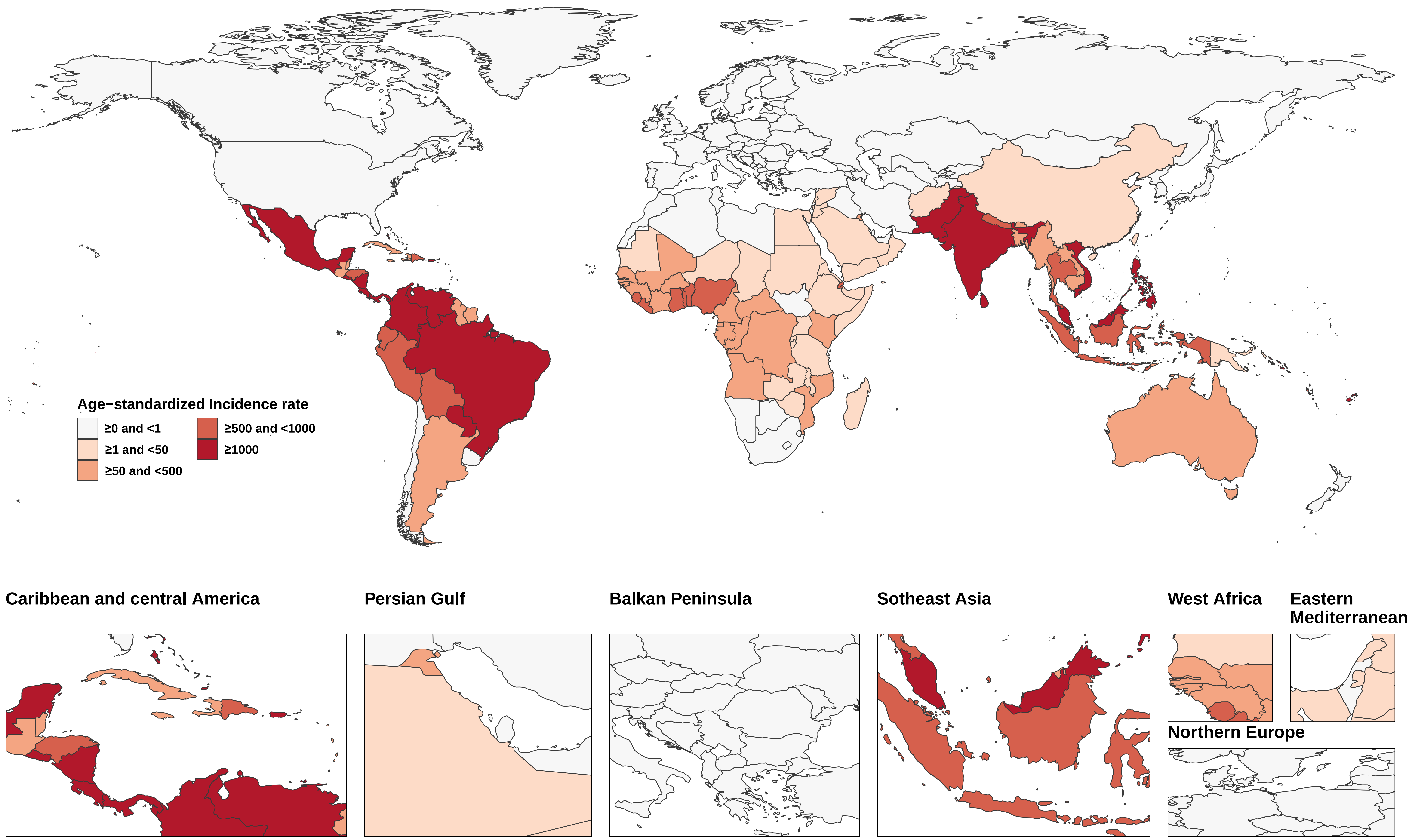
Age−standardized Incidence rate
≥0 and <1
≥1 and <50
≥50 and <500
≥500 and <1000
≥1000
Caribbean and central America
Persian Gulf
Balkan Peninsula
Sotheast Asia
West Africa
Eastern Mediterranean
Northern Europe
