# Supplementary material for: Global burden of dengue from 1990 to 2021: a systematic analysis from the Global Burden of Disease study 2021
Source: Infect Dis Poverty. 2025 Oct 16;14:105. doi: 10.1186/s40249-025-01365-x (PMC12529819; doi:10.1186/s40249-025-01365-x)
Supplement: Supplementary file 3 — Additional file 3: Fig S2. Age-standardized prevalence rates by geographical regions. [file 40249_2025_1365_MOESM3_ESM.pdf]

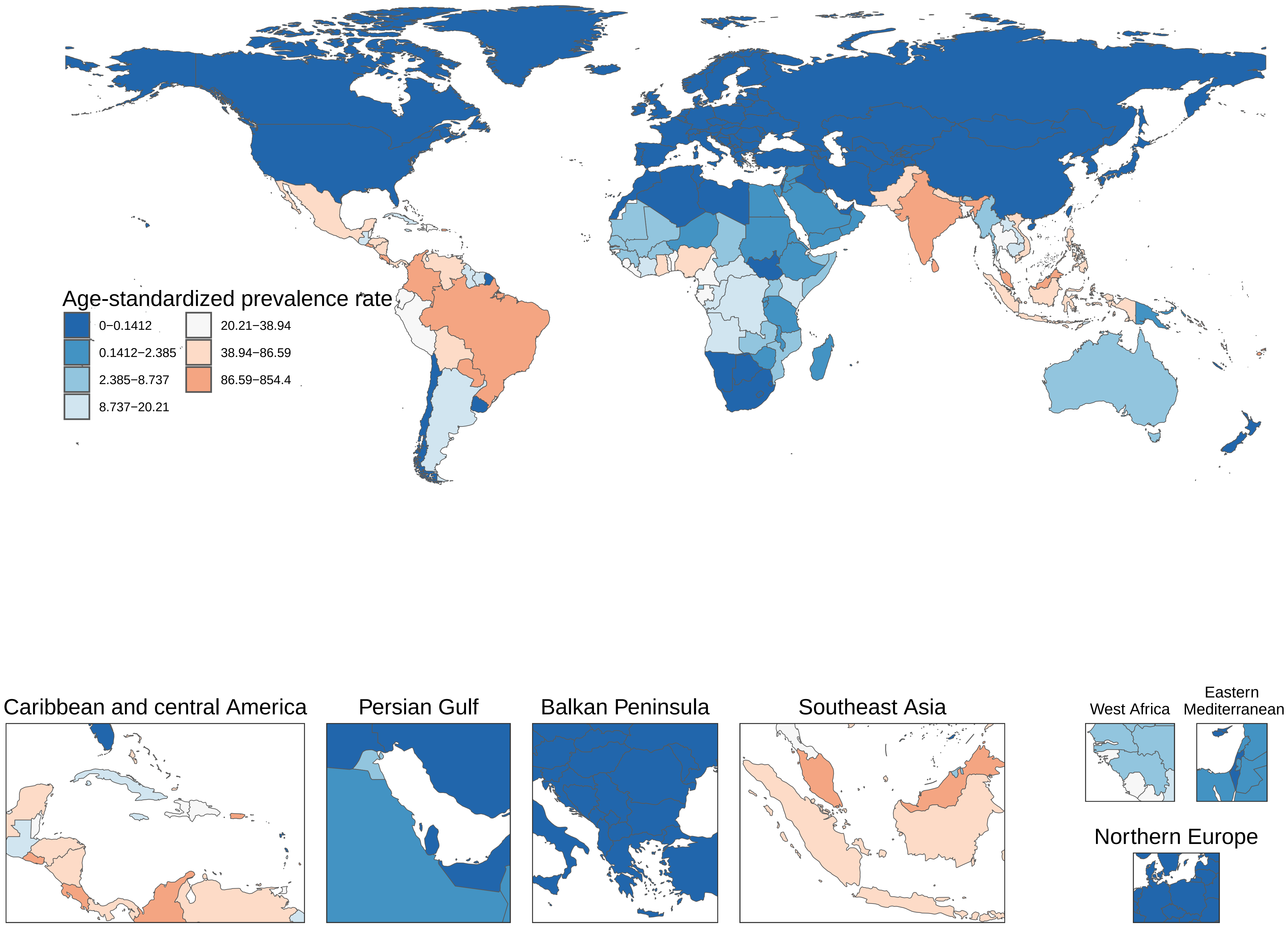

Age-standardized prevalence rate
0−0.1412
0.1412−2.385
2.385−8.737
8.737−20.21
20.21−38.94
38.94−86.59
86.59−854.4
Caribbean and central America
Persian Gulf
Balkan Peninsula
Southeast Asia
West Africa
Eastern Mediterranean
Northern Europe
