# Supplementary figures and images for: Global burden of dengue from 1990 to 2021: a systematic analysis from the Global Burden of Disease study 2021
Source: Infect Dis Poverty. 2025 Oct 16;14:105. doi: 10.1186/s40249-025-01365-x (PMC12529819; doi:10.1186/s40249-025-01365-x)

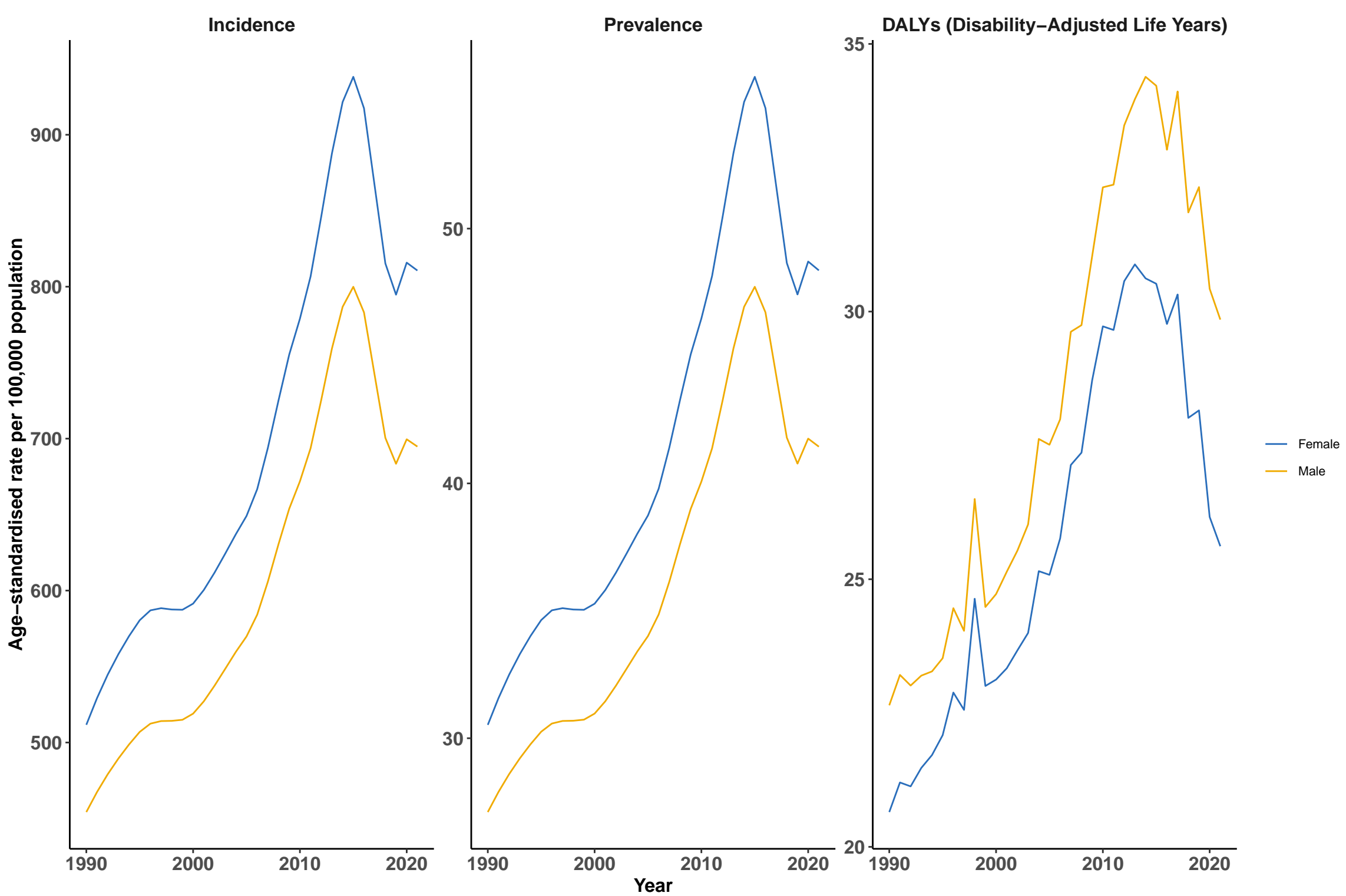

Supplement: Supplementary file 7 — Additional file 7: Fig S3. Temporal trends of incidence, prevalence, and DALYs (both males and females separately) from 1990 to 2020. [file 40249_2025_1365_MOESM7_ESM.pdf]

Age-standardized rate per 100,000 population

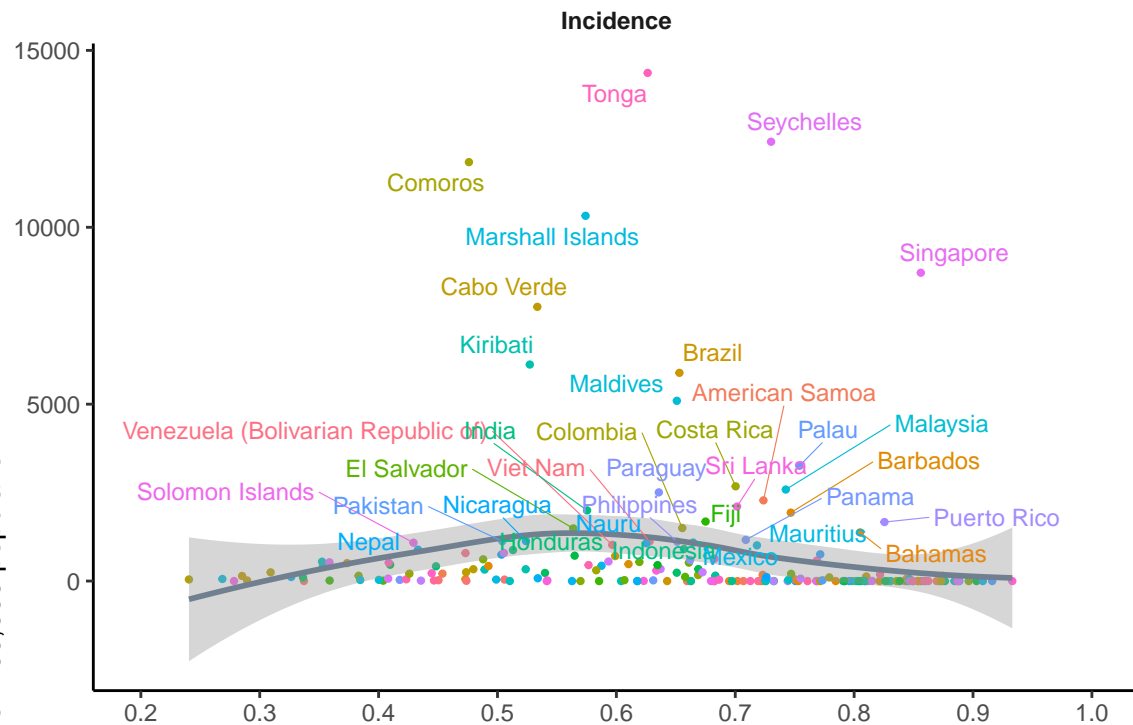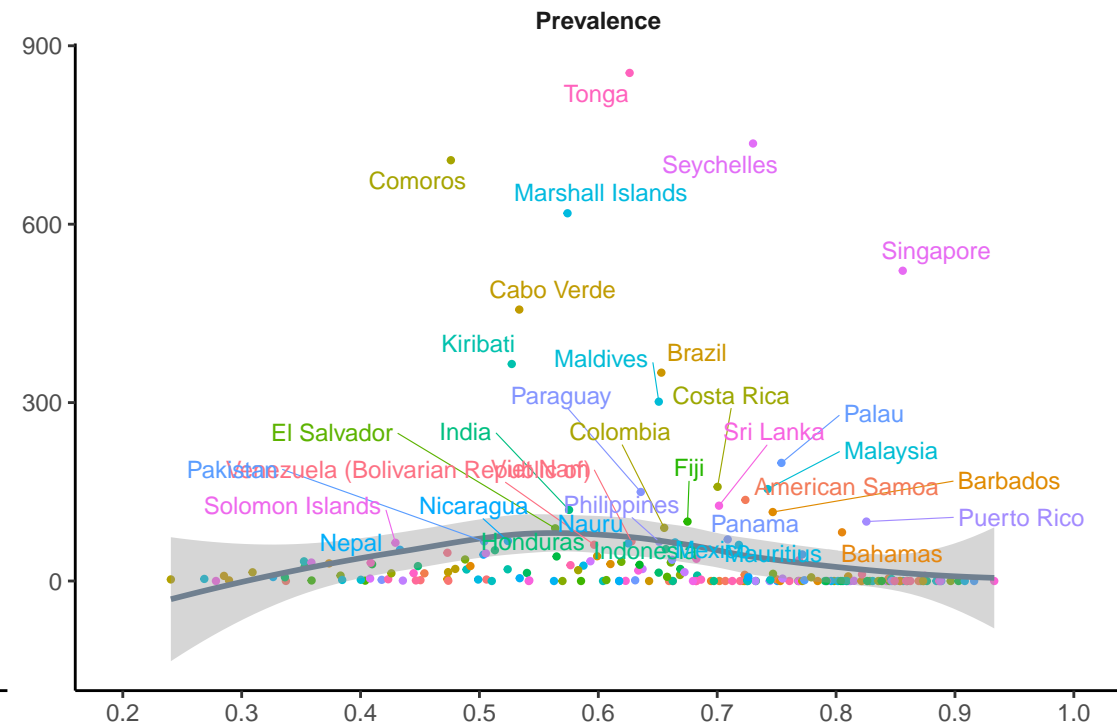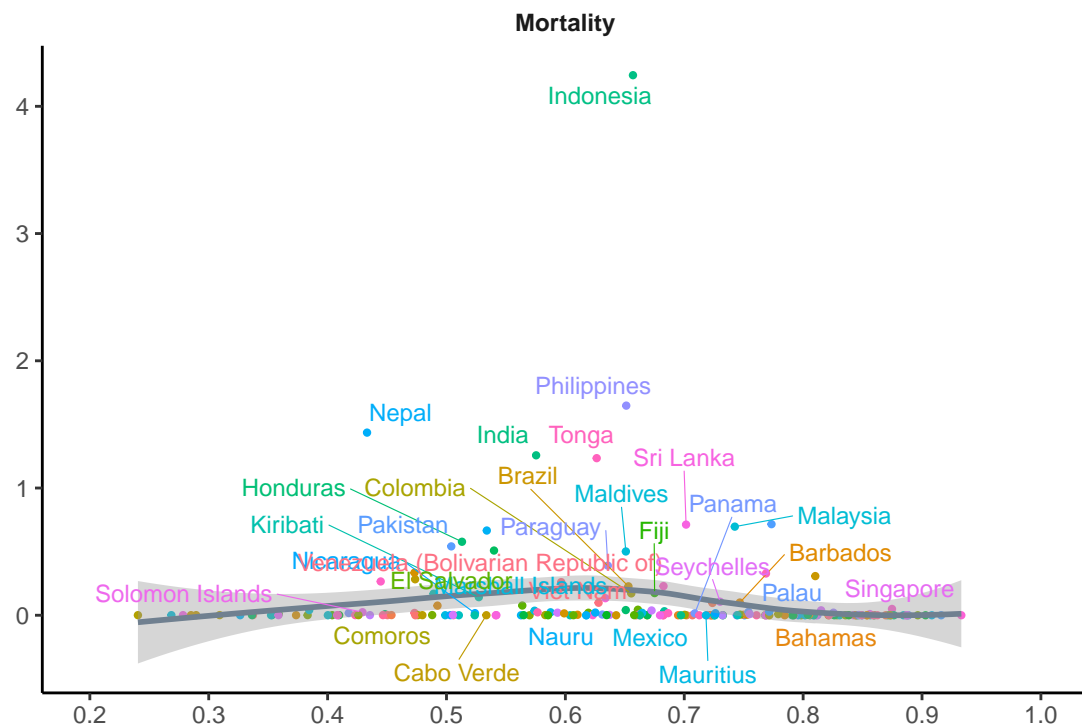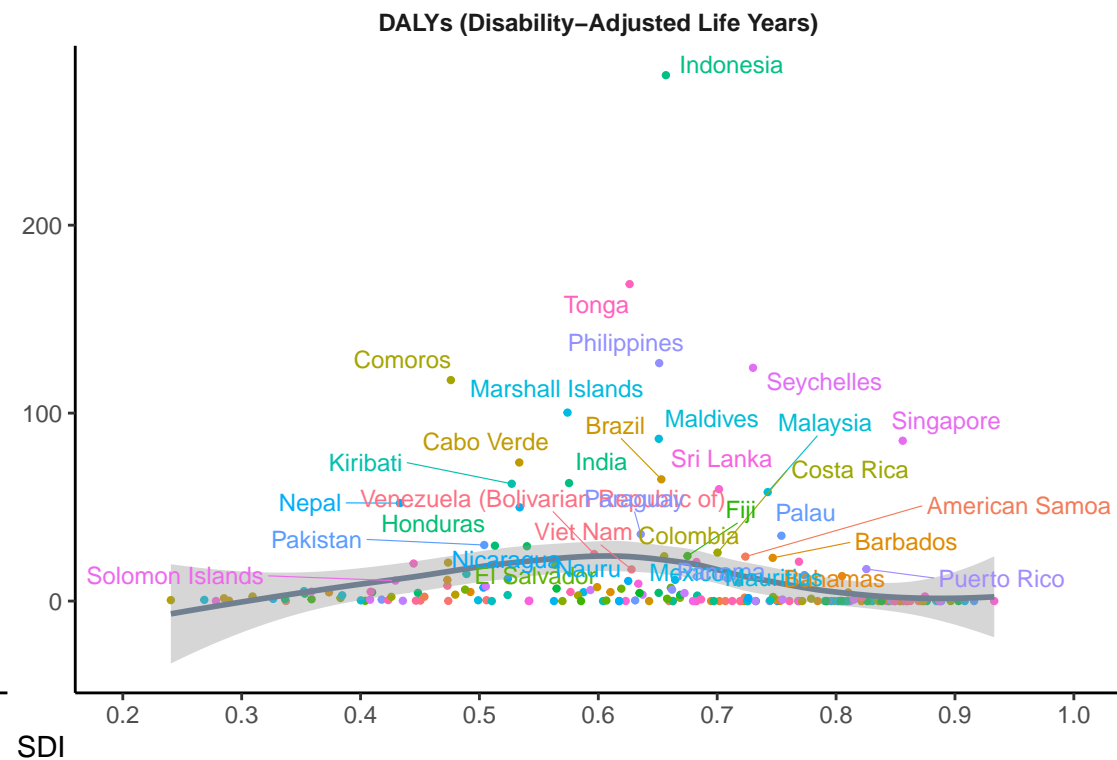

SDI

Supplement: Supplementary file 10 — Additional file 10: Fig S4. Relationship between SDI and Age-Standardized Rates across countries, colored by their respective Socio-demographic Index (SDI). [file 40249_2025_1365_MOESM10_ESM.pdf]
